# Upcycling Calcium Carbonate as an Alternative Filler in Layered Wood Composite Technology

**DOI:** 10.3390/ma18020226

**Published:** 2025-01-07

**Authors:** Julia Dasiewicz, Grzegorz Kowaluk

**Affiliations:** 1 Faculty of Wood Technology, Warsaw University of Life Sciences—SGGW, Nowoursynowska St. 159, 02-787 Warsaw, Poland; s209150@sggw.edu.pl; 2 Department of Technology and Entrepreneurship in Wood Industry, Institute of Wood Sciences and Furniture, Warsaw University of Life Sciences—SGGW, 02-787 Warsaw, Poland

**Keywords:** calcium carbonate, eggshell, layered composite, plywood, filler, upcycling

## Abstract

Chicken eggshells are a useful waste that may be used somewhere rather than being placed in landfills. They are created in poultry hatcheries, the food sector (making pasta, cakes, and egg products), or our homes. In this project, this study aimed to investigate the possibility of producing plywood using a filler in the gluing process in the form of ground eggshells. This study includes the production of plywood with 0, 1, 5, 10, and 20 parts by weight (pbw) of eggshell filler (called E0, E1, E5, E10, and E20, respectively) and one reference variant with rye flour (10 pbw; hereafter called REF10). This research also includes investigating the produced panels’ selected physical and mechanical properties. The results show that chicken eggshells can be used to produce plywood if the right amount of filler is chosen to improve specific mechanical and physical properties. Promising properties were obtained in the determination of the modulus of elasticity under bending (MOE) for samples E5 (11,310 N mm^−2^) and E10 (11,394 N mm^−2^) and modulus of rupture (MOR) for sample E5 (130 N mm^−2^). The results for the internal bond (IB) show that the addition of 5 pbw of filler in the form of ground shells shows good properties with as much as 5.23 N mm^−2^, but still, the reference sample with the addition of filler in the form of rye flour has higher results of 6.22 N mm^−2^. In the test of water absorption of fillers, the absorption of calcium carbonate is 207% and is lower than that of rye flour (224%). For the swelling thickness results, the E10 sample showed the weakest results of 7.6% after 2 h and 8.9% swelling after 24 h.

## 1. Introduction

Solid waste production is around 4 million tons per year worldwide, and most countries produce food waste accounting for between 30 and 45% of the above total solid waste amount [1]. Chicken eggshells are food waste from poultry hatcheries and food production (pasta, cakes, egg products), but also from households. As a by-product, they pose severe economic and environmental problems for producers. As a waste product, eggshells mostly end up in landfills without further processing [2]. However, chicken eggshells, rich in calcium and other minerals, have become valuable in many fields. In agriculture, powdered shells improve the quality of the soil, providing essential nutrients and regulating its pH. As a result, eggshells not only help increase crop yields and improve plant health but can also help remediate contaminated soils, especially those laden with heavy metals such as lead and cadmium [3]. Studies show that using eggshell powder, such as mustard, positively affects the growth of plants by increasing plant height and biomass [4]. In the food industry, shell powder enriches products with calcium, but its addition requires caution due to potential effects on taste and texture. In the chemical industry, on the other hand, shells are used as catalysts in biofuel production, as adsorbents for wastewater treatment, and as construction materials. Such diverse uses of eggshells minimize waste and contribute to a more sustainable economy [3]. The largest egg-producing countries in Europe are France, Germany, Spain, Italy, and Poland [5]. In 2021, approximately 11 billion eggs (consumer and hatching) were produced in Poland [6], and around 50,000 tons of eggshells are produced worldwide each year [7]. Eggshells consist mainly of calcium carbonate in the form of ca, for example, lcite (94%). In addition to CaCO_3_, the shell also contains other inorganic compounds, for example, calcium phosphate (<1%), magnesium carbonate (<1%), and silicon oxide (<1%). Approximately 4% of the remaining shell mass comprises various collagens, polysaccharides, fatty acids, and water [8,9].

Waste in the form of eggshells has begun to be used in various fields conducting various studies. One such study by [10] uses eggshells as a biofilter for acrylonitrile butadiene rubber (NBR) latex film for glove applications. The latex was cured at 80 °C for 120 min while the film was being made. All composite films have higher tensile strength and Young’s modulus than the control film. With increasing filler, the resistance to swelling and chemical deterioration rises. The findings indicate significant potential for adding eggshell filler to NBR in Type I and II manufacture.

On the other hand, in a study by [11], they used eggshell powder instead of cement to form columns out of a reinforced concrete flat slab. Four distinct concrete mixes comprising 0, 5, 10, and 15% eggshell powder were used to cast the flat reinforced concrete slabs (S1, S2, S3, and S4) in the four columns. The slabs in punching mode all failed.

In other studies, poplar veneers have been impregnated with calcium carbonate (CaCO_3_) to improve performance [12]. The most significant weight gain following impregnation was 41.4%, while water consumption decreased from 6.82% to 0.94%. The rate and total heat release were modest, and the ignition time was extended. According to experimental results, CaCO_3_ improves poplar veneers’ physical and mechanical characteristics and fire resistance.

The impact of adding TiO_2_ and CaCO_3_ (chalk) to phenolic resin on the glue line’s color and the characteristics of waterproof plywood were examined in a study by [13]. The investigation examined the impacts of TiO_2_ and CaCO_3_ on the plywood’s mechanical qualities and water resistance, as well as the degree of brightening of the cured resin samples based on the CIE L*a*b* model. Experiments have demonstrated that a 50% substitution of TiO_2_ for chalk filler is ideal, allowing for the production of plywood with the necessary characteristics, including a vivid glue line color and the lowest possible material costs.

So far, the production of plywood has used a variety of bonding techniques and forms. For example, the utilization of fiber matting [14] as a sustainable filler and waste material from furniture manufacturing has greatly enhanced the screw withdrawal strength and internal bond. In terms of formaldehyde emissions, fillers in the form of ground bark from birch, beech, maple, pine, and spruce [15] improved the findings, showing decreases for birch (B-1), beech (B-2), maple (B-3), and pine bark (B-4). Furthermore, formaldehyde emissions were shown to increase specifically for spruce bark. *Camellia oleifera* shell powder and palm kernel powder [16] enhanced melamine-urea-formaldehyde (MUF)’s bonding performance in plywood production; modified buckwheat hull [17] as a filler for urea-formaldehyde resin resulted in better bond quality and decreased delamination. In addition to lowering formaldehyde emissions and saving over 40% on adhesive expenses, they had a binding strength comparable to industrial powder.

In the production of plywood, various starches such as chestnut flour, for example, have so far been used as a filler [18], including flour from ground banana peels [19], ground hazelnut shell [20], and coffee bean residues [21]. In the case of chestnut starch-based filler in plywood technology, it shows excellent elasticity and bending modulus properties when used in appropriately selected amounts. Also, flour from waste banana peels can be a valuable alternative filler in plywood technology. In contrast, using post-extraction residues from coffee beans resulted in reduced or no property improvement.

The type of filler used in plywood significantly affects its density and overall properties. Different fillers, including organic flours and inorganic minerals, alter plywood’s physical and mechanical properties, affecting its density through their unique composition and interactions with the adhesive. Different flours (e.g., rye, hemp, and pumpkin) affect UF resin’s viscosity and gel time, affecting the density of the plywood produced. For example, pumpkin flour provided better mechanical properties and bond quality [22]. Adding mineral fillers, including sepiolite and calcium carbonate, may increase density by adding more volume. For instance, using sepiolite instead of wheat flour significantly increased the wet shear strength of plywood [23]. While mineral fillers at low concentrations (up to 10%) barely affect density, greater concentrations can cause mechanical characteristics to deteriorate because of decreased fiber-to-fiber bonding [24,25]. The bonding interface in plywood refers to the region where adhesive interacts with wood veneers, establishing structural and mechanical integrity. Characterizing this interface is essential for understanding and optimizing performance parameters such as strength, durability, and environmental resistance. The adhesive, commonly urea-UF resin or modified forms, penetrates the porous wood structure, forming a network of mechanical interlocks and chemical bonds. The presence of fillers, such as calcium carbonate or organic materials like rye flour, significantly affects the microstructure and performance of this interface. Fillers modify the viscosity and curing behavior of the adhesive, impacting penetration depth and bond-line thickness.

Therefore, this project aimed to investigate the possibility of producing plywood using a filler in the bonding mass of ground eggshells. During the study, the plywood was created with different amounts of filler (0, 1, 5, 10, and 20 pbw) in the bonding mass. Then, the selected mechanical and physical properties of the composites that were made were investigated. The study presents an innovative approach to utilizing waste chicken eggshells as an eco-friendly filler in plywood production. By incorporating varying proportions of ground eggshells into the bonding mass, the research explores their impact on mechanical and physical properties, achieving optimal results with 5–10 parts by weight. This pioneering use of sustainable waste material highlights its potential to replace conventional fillers, contributing to waste upcycling and promoting environmental sustainability in wood composite manufacturing.

## 2. Materials and Methods

### 2.1. Materials

**Figure 1:**
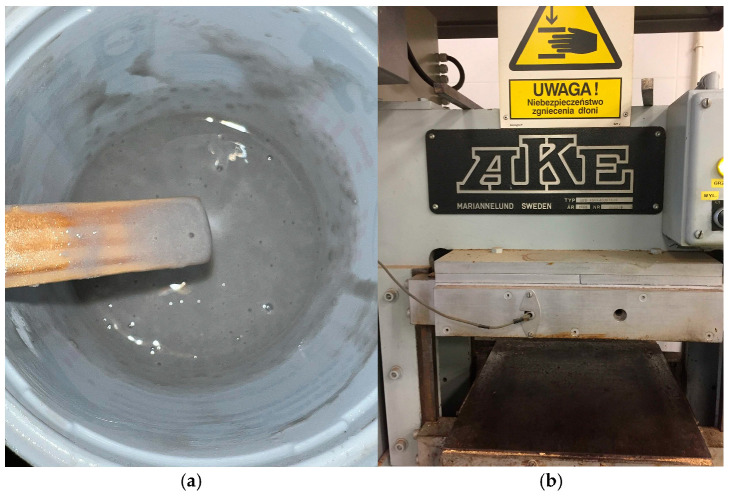
The E10 bonding mass before spreading on the veneer (**a**), and the hot press used to press the composites (**b**).

Materials used for the test samples included the following: beech veneer (*Fagus sylvatica* L.); flat-cut, with an average thickness of 0.62 mm; eggshell (ES) in powdered form as a filler (ground, laboratory-made; grain size < 0.125 mm; 2.8% moisture content); rye flour, commercially available, as filler in the reference samples; hardener −2% dry weight of ammonium nitrate (NH_4_NO_3_) (CAS 6484-52-2; WARCHEM Sp. z o.o., Trakt Brzeski St. 167, 05-077 Zakręt, Poland) aqueous solution; industrial urea formaldehyde resin (UF) (Silekol Sp. z o.o., Kędzierzyn-Koźle, Poland), ca. 65% solid content, mean 98 s curing time in 100 °C (with following nominal ratios of components: 100:4:10:5, resin/hardener water solution/filler (rye flour)/distilled water, respectively); and distilled water. The viscosity [26] of the reference bonding mass, characterized above, was about 440 mPa s. An example of the prepared bonding mass (E10) before spreading onto the veneer surface has been presented in Figure 1a. The following viscosity of the remaining varieties of the tested bonding mass have been registered: 260, 268, 315, 420, and 480 mPa s, respectively, for E0, E1, E5, E10, and E20.

### 2.2. Samples Preparation

The three-layer composite (plywood) was made using powdered eggshells as a filler in the bonding process. The glue mixture of 180 g m^−2^ was uniformly applied to the veneers using a brush. The samples were pressed through 5 min of high-temperature pressing (140 °C press temperature and 1 MPa press unit pressure) (Figure 1b). After pressing, the samples were conditioned for 7 days at 20 °C and 65% ambient air humidity to stabilize the sample’s mass. The varieties of the produced composites with their codes are presented in Table 1.

### 2.3. Characterization of the Manufactured Panels

**Figure 2:**
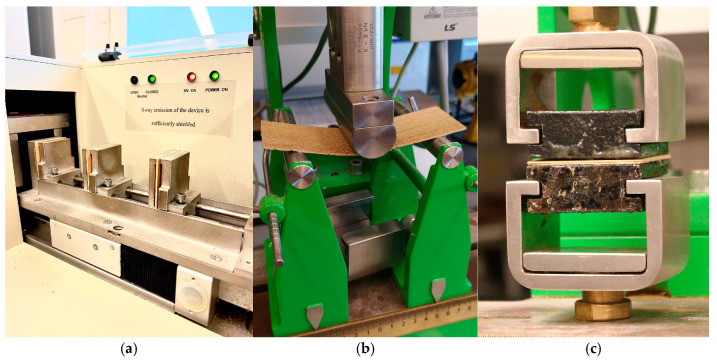
The measurement of the density profile (**a**), the bending test (**b**), and the internal bond test (**c**) of the produced samples.

**Figure 3:**
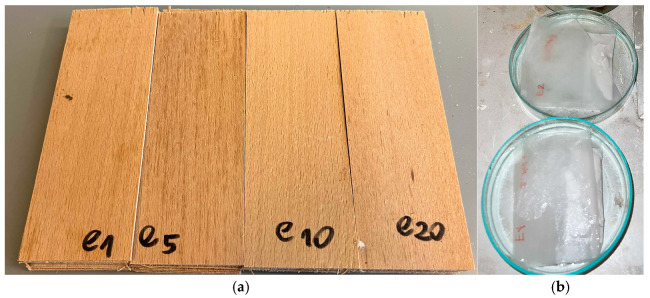
The prepared samples before bending (**a**), and the filler samples during the water absorption test (**b**).

A DA-X measurement instrument (Fagus-GreCon Greten GmbH and Co. KG, Alfeld/Hannover, Germany) was used to analyze the materials’ density profiles (Figure 2a). The measurement, based on direct X-ray scanning densitometry, was performed throughout the whole thickness of the panel at a speed of 0.1 mm s^−1^ with a sample step of 0.02 mm. The nominal measurements of 50 mm × 50 mm were used to cut the samples. Three samples were used to analyze the density profile of each kind of composite. As a result, one representative profile from each panel variation evaluated was shown following the first examination. The images of the cross-cuts of the investigated plywood samples were taken with a Nikon SMZ 1500 (Kabushiki-gaisha Nikon, Minato, Tokyo, Japan) optical microscope. According to the standard [27], mechanical tests, including the modulus of elasticity (MOE) and modulus of rupture (MOR), were performed (Figure 2b) using computer-controlled universal testing equipment (Research and Development Center of the Wood-Based Panels Industry Sp. z o.o., Czarna Woda, Poland) on the samples with dimensions of 150 mm length (along the fibers) and 50 mm width (Figure 3a). An internal bond (IB) strength test was also conducted [28] on samples 50 × 50 mm on the same testing machine (Figure 2c). The analysis was performed for all variations, and the thickness swelling during water immersion was calculated using the standard for particleboard and fiberboard [29] on samples 50 × 50 mm. The identical samples were then subjected to a water absorption test.

Additionally, filter paper containers were used to assess the filler’s ability to absorb water. Each container received 1–2 g of eggshell flour or rye flour. Two samples were used to evaluate each filler. Each sample was soaked for 10 min at around 20 °C ± 1 °C in demineralized water (Figure 3b), and then it was allowed to dry for 10 min before its weight was determined.

### 2.4. Statistical Analysis

An analysis of variance (ANOVA) and t-test calculations were used to test (α = 0.05) for significant differences between factors and levels, and where appropriate, using IBM SPSS statistic base (IBM, SPSS 20, Armonk, NY, USA). The Results and Discussion paragraph gives the statistically significant differences in achieved results whenever the data were evaluated.

## 3. Results and Discussion

### 3.1. Density Profile

**Figure 4:**
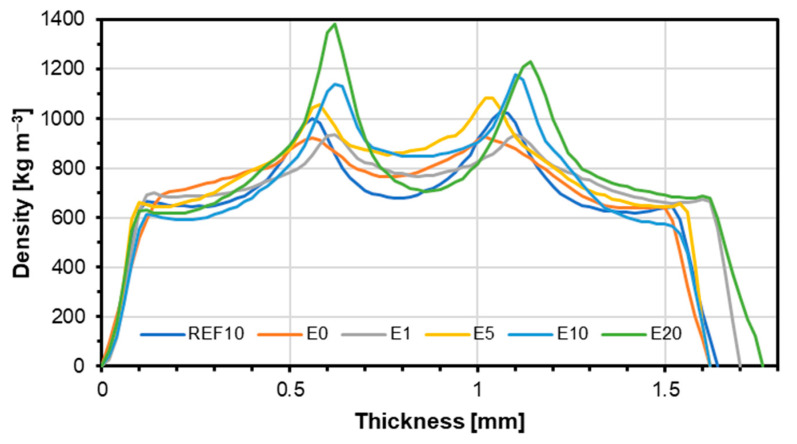
The density profiles of tested samples.

**Figure 5:**
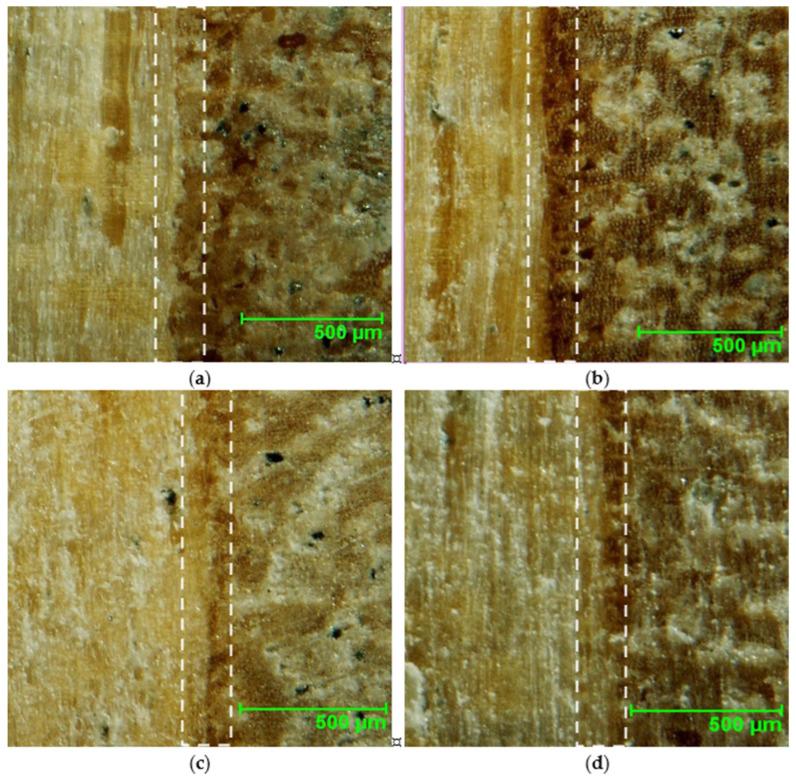
The examples of the bonding lines for samples E0 (**a**), E5 (**b**), E20 (**c**), and REF10 (**d**) (dashed block shows the veneers interface zone).

The results for the density profile are shown in Figure 4. As can be seen from the graph, as the eggshell content increases, the density in the bonding lines increases, so the highest density in the bonding line is shown by the sample with the highest ES addition (E20) at 1383 kg m^−3^, while the sample shows the lowest density without any filler added (E0) and its density is 921 kg m^−3^. In the inner layer, sample E5 has the highest density (853 kg m^−3^) and sample REF10 has the lowest density (691 kg m^−3^). The relative difference between the density of the inner layer of E5 and REF10 is about 26% and between E5 and E0 is about 10%. The high density of the inner layer of the E5 sample (Figure 2b) can be due to the specific accumulation of materials and technological factors. Based on the materials’ factors, the bonded wood surface and filler functionality that tunes the migration of the binder to the veneer structure should be mentioned. The technological features that can affect the density of the inner layer of the tested plywood can be the pressing time and curing of the bonding mass. This means the 5 pbw filler content leads to proper penetration of the veneers, simultaneously allowing for the creation of the bonding line [30]. Increasing filler content limited the veneer impregnation, as can be seen in Figure 4. It is also worth adding that with the increasing filler amount, the peaks around the bonding lines become narrower. That is evidence that the ES is a required bonding mass filler, reducing wood impregnation by bonding mass. Pictures of the cross-section of the tested samples for variants E0, E5, E20, and REF10 have been presented in Figure 5.

A similar density profile for plywood was shown in the study of [19], which uses a filler of ground banana peels to produce plywood. The inner layer with the lowest filler value, BMIX5, shows the lowest density; sample BO10 obtained the highest density in this layer. In the outer layers, the BO20 sample achieved the highest density and the BO5 sample the lowest. In addition, increasing filler content reduces the penetration of the veneer by the binder, as indicated by narrower bond line peaks. The test showed reduced veneer penetration through the binder with increasing filler content.

In contrast, the density profile is slightly different in a study of cellophane-glued plywood [31]. In the ash veneer samples (A0, A1, and A2), the binder density reaches the highest values as it is over 1100 kg m^−3^ for thicknesses of around 0.6 mm and 1.18 mm. Using a thermoplastic regenerated cellulose binder affects the thickness and density distribution of the composite. The binder impregnates the veneers, and this is visible because there is a smooth transition of the veneer density to the higher density of the binder. As the pressing time increases, the thickness of the composites decreases while the density increases significantly. Using a thermoplastic binder based on regenerated cellulose made it possible to achieve remarkable results in wood compaction. The binder, melting and penetrating the wood structure, significantly increased its density, exceeding 55%. The results of density profile studies confirmed this fact. Accordingly, supersaturated veneers through cellophane improved the density of veneers by increasing it up to 800 kg m^−3^ at the outer veneer for a beech sample bonded with cellophane and pressed for a shorter time (B1). These results show a higher density of the outer veneers in the plywood than using calcium carbonate, where the veneers were not saturated with the binder and the highest density with the outer veneer was 700 kg m^−3^ (E20). In contrast, the density of the binder came out higher for the sample using calcium carbonate because as much as 1383 kg m^−3^ for sample E20, and for samples using cellophane as a binder, the highest density was 1100 kg m^−3^ for sample B1.

Increased filler content typically narrows bond-line peaks in density profiles, suggesting reduced adhesive penetration and enhanced bonding efficiency at the interface [32]. What is more, the eggshell powder filler, primarily composed of CaCO_3_, which accounts for approximately 94% of its mass [9], having an average density of about 2.7 g cm^−3^ [33], enhances the density of the bond line, improving the stiffness and load-bearing capacity of the plywood.

### 3.2. Determination of Modulus of Elasticity in Bending and of Bending Strength

**Figure 6:**
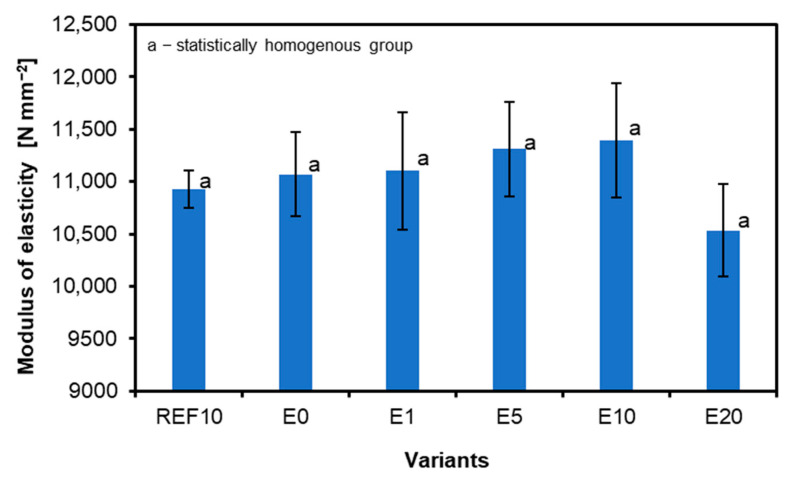
Modulus of elasticity of tested samples.

The results for the modulus of elasticity in bending are shown in Figure 6. An increase in the addition of eggshell increases the modulus of elasticity, but only up to sample E10, which shows the highest properties (11,394 N mm^−2^), as a higher filler addition decreases the properties and thus sample E20 shows the lowest modulus of elasticity (10,534 N mm^−2^). Samples E0, E1, E5, and E10 have a higher modulus of elasticity than the reference sample (REF10), which has a MOE of 10,926 N mm^−2^. Regarding REF10 and E0, the highest E10 MOE value is higher at 4.3% and 2.9%, respectively. No statistically significant differences have been found among the MOE average values. The relatively high scatter of results, displayed on the plot by error bars, can be attributed to the manual bonding mass spread imperfection and the wood veneers’ local, natural inhomogeneity.

In their study, Kawalerczyk et al. [22] added the following to UF resin in producing laminated wood: rye flour, hemp flour, coconut flour, rice flour, and pumpkin flour to UF resin. For every 100 g of resin dry weight, 15 g of flour and 15 g of water were added. The flour type employed considerably impacted the resin mixture’s viscosity, solid content, and gelation time. The gelation process was lengthened by including rye and pumpkin flours; however, the resinous composition enhanced with rice flour lacked the viscosity required to produce plywood. The data collected chiefly agreed with the adhesive quality assessment’s findings. The modulus of elasticity varied for each type of filler. MOE properties were investigated using both longitudinal and perpendicular orientations. The largest MOE in the longitudinal direction is plywood manufactured with rye flour (14,000 N mm^−2^) and pumpkin flour (14,500 N mm^−2^). However, coconut flour produced a lower MOE (10,100 N mm^−2^). These results were better than when using calcium carbonate filler and better than when applying the perpendicular direction. Such results may have been due to the different chemical composition of the filler, which affected its hydrophilic properties, reactivity, and overall material properties.

In a study completed by [15], various types of bark (birch B-1, beech B-2, maple B-3, pine B-4, and spruce B-5) were used as a filler (20 pbw.) in the production of UF resin-based laminated wood. The MOE characteristics were studied in both the perpendicular and longitudinal directions. In this study, MOE also came out higher for the longitudinal than the perpendicular direction. Thus, the direction in which a particular test is conducted is fundamental. The MOE results in the perpendicular direction for the REF, B-1, B-2, B-3, and B-4 variants were statistically similar around 1000 N mm^−2^.

In contrast, variant B-5 (750 N mm^−2^) significantly deteriorated this parameter. The differences were noticeable for MOE in the longitudinal direction, and statistically significant differences were observed. The best results came out for variants B-1 (12,800 N mm^−2^) and B-2 (12,600 N mm^−2^). In contrast, the lowest result came out for sample B-4, amounting to only 10,500 N mm^−2^. Comparing these results with those of the calcium carbonate samples, better results were obtained for the bark samples. Scientific studies show that exceeding the threshold of 15% additive in the resin leads to a significant deterioration of its fluidity, manifesting in a substantial increase in viscosity [32,34,35]. Similar observations were made by Réh et al. [36], who found that adding 20% bark particles (in the dry weight of the resin) leads to an increase in the viscosity of the resin. High viscosity can make it challenging to spread the adhesive evenly, harming the bond’s quality. The effect of a high content of these compounds on mechanical properties is still debated in the literature, and the explanations given are often contradictory. For example, on the one hand, a high content of these compounds can lead to improved mechanical properties [36]. On the other hand, a high proportion of these compounds lowers mechanical properties while increasing viscosity, translating into resin life and affecting intermolecular crosslinking [37,38]. Studies confirming a reduction in MOE scores were observed by Nemli et al. [39]. Including nanocellulose at specific concentrations improved bonding and plywood quality, but higher amounts had a negative effect [40]. Furthermore, carbon nanotube fillers substantially increased the viscosity of electrically conductive adhesives, highlighting their impact on adhesive properties [41].

The type of glue, the kind of wood, and the joining technique all impact the test specimens’ MOE value. The kind of wood has a significant impact on the outcomes as well [42,43]

**Figure 7:**
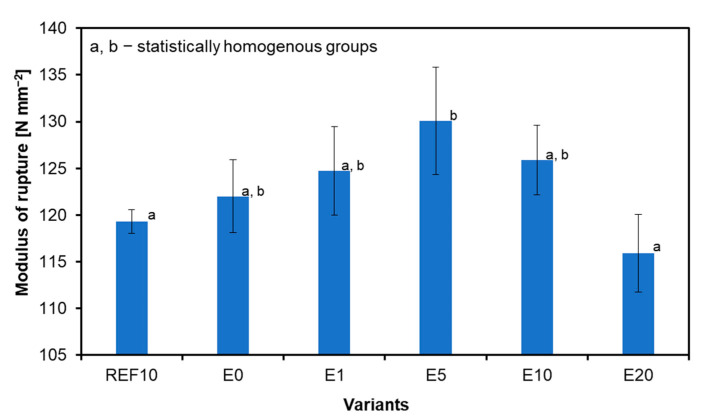
Modulus of rupture of tested samples.

**Figure 8:**
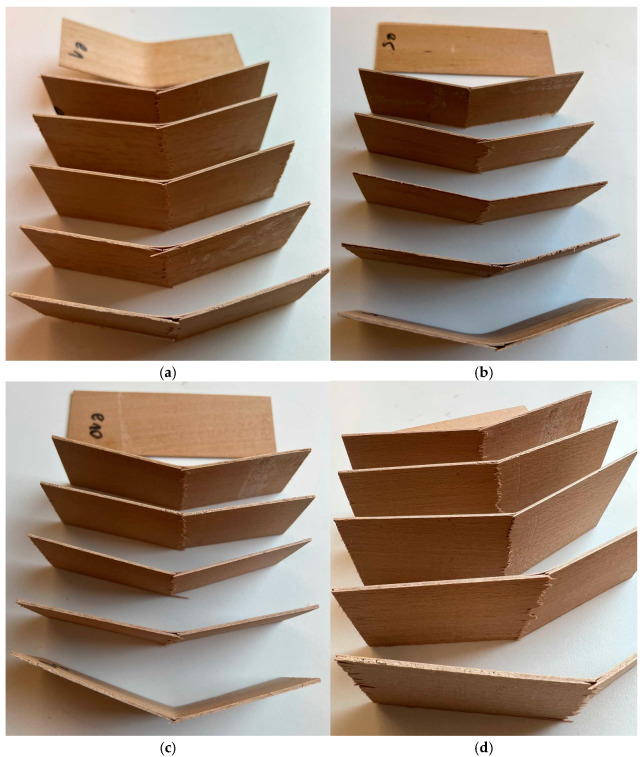
The selected examples of the tested composites after the bending test: E1 (**a**), E5 (**b**), E10 (**c**), and E20 (**d**).

The results for the MOR are shown in Figure 7. Sample E5 (130 N mm^−2^) indicates the highest modulus of rupture, and the lowest is demonstrated by the samples with the highest addition of ES (E20) at 116 N mm^−2^. Samples E0, E5, and E10 show a higher modulus of rupture than the reference sample (REF10) of 119 N mm^−2^. Regarding REF10 and E0, the highest MOR value of E5 is higher for 9.0% and 6.6%, respectively. The only statistically significant differences have been found among the MOR average values between E5 when referring to remaining composites. The examples of the selected, tested composites after the bending test are shown in Figure 8.

Research on plywood made using bark particles of different sizes as filler for urea-formaldehyde resin [44] showed that bark powder with a dimensional fraction of 0.315 mm could be the substitution for rye flour in the production of plywood with comparable mechanical qualities. For this sample, the results are better than those of the control sample and allow for uniform application and a bond with the highest strength values. Other samples showed significantly lower properties.

Other studies have been carried out by [36], who explored the impact of using birch bark as a filler for plywood. They used 10, 15, and 20 parts by weight of birch filler for every 100 parts of UF resin. The sample with an average amount of bark (15 pbw) showed the highest MOR value.

### 3.3. Internal Bond

**Figure 9:**
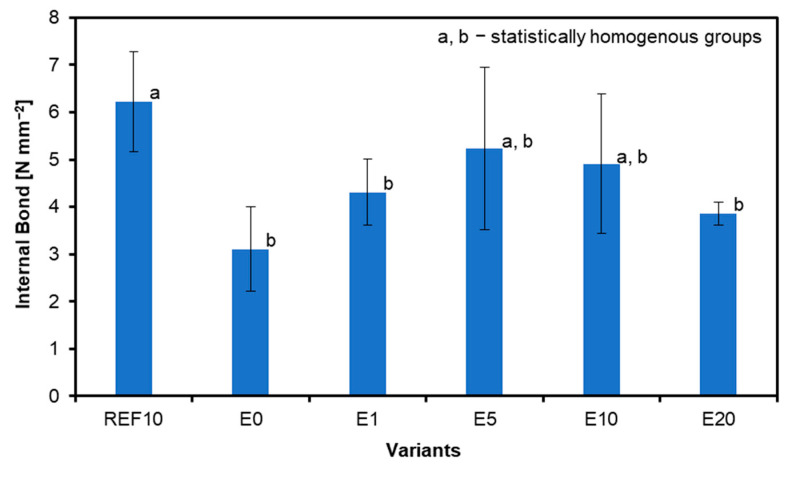
Internal bond of tested samples.

The results for the internal bond are shown in Figure 9. The reference sample (REF10) demonstrates the highest IB at 6.22 N mm^−2^ and the lowest IB without any filler additive (E0) at 3.11 N mm^−2^. The IB increases from sample E0 (3.11 N mm^−2^) to E5 (5.23 N mm^−2^) and then gradually decreases to 4.91 N mm^−2^ (E10) and 3.85 N mm^−2^ (E20). The relative difference between the REF10 IB value and the highest IB of variants with eggshell filler, E5, is about 19%. The only statistically significant differences among the IB average values between E0 and REF10 panels have been found. No significant visual differences among the tested samples have been found in the case of the destruction image.

According to the mechanical model, component size, resin content, and product density all impact IB strength. Better IB strength is correlated with higher density and resin content. Density variations can cause a substantial weakening of the connection, with low-density regions detaching earlier [45]. Various mechanical tests, including edgewise shear and interlaminar tests, shed light on the IB quality of plywood and other wood-based panels [46]. The accuracy of IB evaluations can be improved by using a uniform specimen size for testing, producing more consistent and trustworthy findings [47]. The cost-effectiveness of adhesive formulas and production techniques should be considered, as this might affect the overall quality of the product and its viability in the market, even if internal bond strength is essential for enhancing plywood performance [48].

### 3.4. Filler Water Absorption

**Figure 10:**
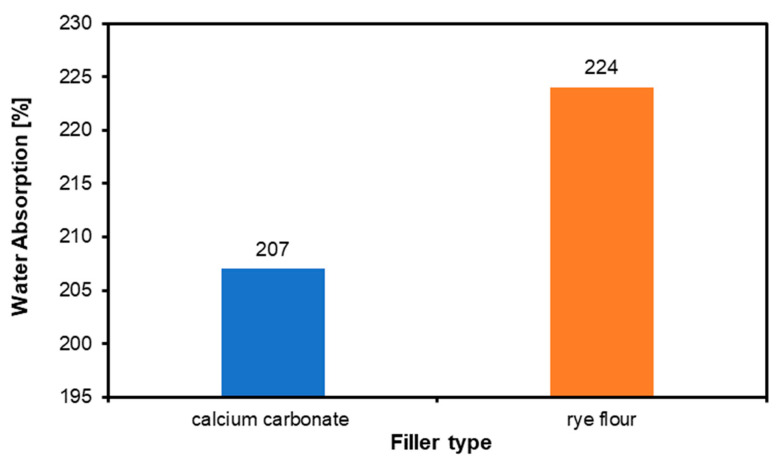
Water absorption of calcium carbonate and rye flour fillers.

The results for the absorption are shown in Figure 10. The absorption of calcium carbonate is 207%, which is lower than the absorption of rye flour, 224%.

In the research of Dasiewicz and Wronka [18], chestnut flour was used as a filler in plywood production. Chestnut flour showed a water absorption ability of 228%. So, that absorption level is higher than calcium carbonate’s but similar to rye flour’s. On the other hand, hazelnut shells had an adsorption capacity of up to 419%. Thermally modified hazelnut shell flour absorbed the least water (79%), while chemically modified hazelnut shell flour absorbed 186% [20]. In the case of chestnut flour plywood, a clear correlation was observed between reduced filler adsorption (228%) and an increase in MOE and MOR. These results are significantly better than those of plywood with hazelnut shell filler. An analysis of the data indicates that both too much (419%) and too little (79%) saturation of the hazelnut shell filler leads to the deterioration of the mechanical properties of the plywood. Therefore, it can be concluded that the optimal properties of plywood depend on the appropriate saturation level of the filler, which can be different for each type of filler.

Wojciechowska and Kowaluk [19] have used ground banana peels as a filler in plywood production. They also used rye flour for a reference sample; the adsorption result was similar, 224%. The sample with the outside of the banana peel had an absorbance as high as 531%. The banana peel core sample had a lower absorbance of 330%. The final sample for evaluation had an absorbance of 412% and was prepared from a banana peel blend flour. In all cases, the adsorption was higher than that of the reference rye flour. The best result of the plywood properties for MOE came out with the sample using 5 pbw banana core skin peel flour as a filler (BC5), but also with the same filler; all samples showed higher properties than the samples where banana outer skin peel flour was used as a filler. The MOR results of the plywood came out best for all the samples with 10 pbw. of filler, and based on this, the best results came out with the sample where the absorbency of the filler was 224% adsorption; slightly lower results came out for the fillers with 330% and 412% adsorption. The weakest results came out for the sample with the filler with the highest absorbency (531%). Therefore, it can be concluded that the reduced adsorption of fillers positively affected the MOE and MOR of the plywood. This observation is confirmed by the results of samples with calcium carbonate filler, which showed lower absorption (207%) and significantly higher MOE and MOR values compared to samples with banana filler.

Water absorption is affected by many factors, including particle or dust size, substance content, and soak time [49,50,51].

### 3.5. Swelling in Thickness and Water Absorption

**Figure 11:**
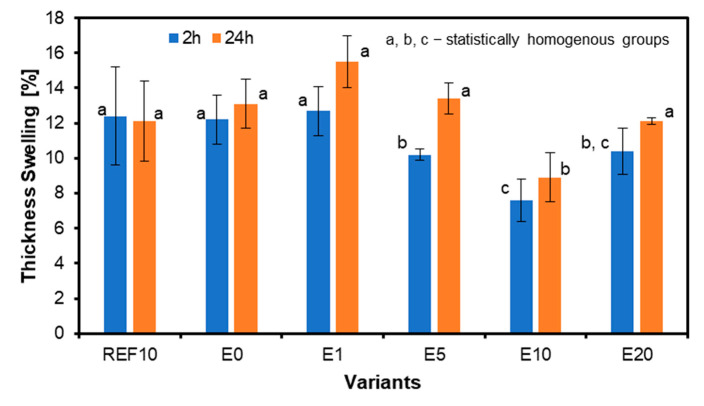
Swelling of the thickness of the plywood with different eggshell filler content.

The results for the swelling in thickness are shown in Figure 11. Swelling in thickness decreases with increasing eggshell filler content. There is a slight difference between 2 h and 24 h soaking. After 2 h, sample E1 shows the highest TS, and sample E10 the lowest. After 24 h, analogously, the TS after 2 h also shows the highest in sample E1 and the weakest in sample E10. The reference samples after 2 h and 24 h show minimal TS differences. The relative difference between the highest thickness swelling after 24 h of sample E1, referred to as REF10, is 28.1%. The only statistically significant differences in average values of 2 h TS have been found between E5 when referred to remaining panels except E20. For 24 h TS results, statistically significant differences have been found for E5 when referred to E10 and E20 panels and E10 when referred to E0, E1, and E20.

TS on produced plywood reinforced with nanoparticles and kenaf fiber was examined during the investigation [52]. The samples with kenaf and nanosilica (KNS) (3.85%) had the lowest TS values (24 h), and the UF resin blank had the most significant TS values (24 h). The chemical connections between the kenaf fibers and the wood layers are strengthened using kenaf fibers with higher hemicellulose content. Consequently, there is less chance that water will seep into the reinforced panel construction.

On the other hand, studies on the fibreboard with different types of anti-adhesive (release) agents investigated TS [53]. The tests show that as the content of both adhesives “A” and “B” increases, the TS decreases after 2 h and 24 h. After 24 h, the highest TS is shown by sample “B” with a 10 g m^−2^ release agent spread (34.1%).

A study [54] investigated TS on panels produced from giant reeds glued with unmodified starch. As the adhesive content grew, TS values increased as well. The highest TS values were found in the adhesive-free particleboards. After two hours of immersion, the samples’ average thickness swelled by 18 to 61%. The TS results for the 24 h immersion varied from 18% to 80%.

**Figure 12:**
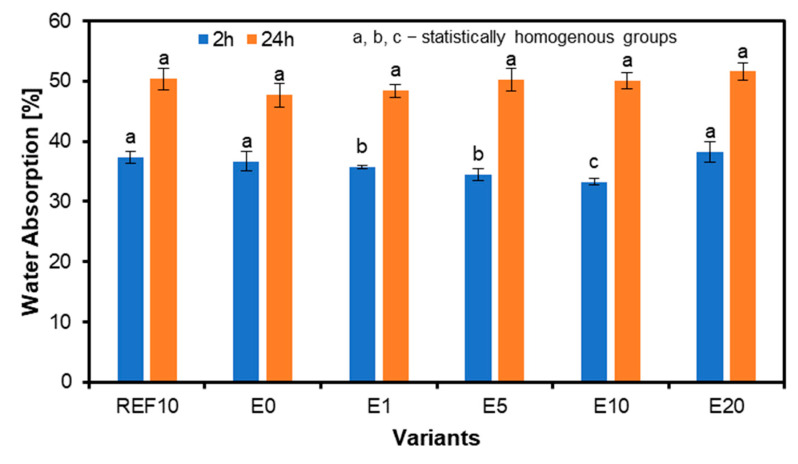
The water absorption of the plywood with different eggshell filler content.

The results for the water absorption are shown in Figure 12. Water absorption after 2 h as well as after 24 h increases consistently. Sample E20 shows the highest WA after 2 h, while sample E0 shows the lowest. After 24 h, the WA is highest for sample E20 and lowest for sample E10. Samples with a filler content of 10 reference pbw (REF10) and those using eggshells (E10) show the same WA after 2 h. After 24 h, the difference is less significant than REF10, 37.3%, and E10, 50.4%. The relative difference between the highest water absorption after 24 h of sample E20, referred to as REF10, is 2.4%. The statistically significant differences in average values of 2 h WA were found between REF10 when referring to the remaining panels, except for E0 and E20. For 24 h WA results, statistically significant differences have been found for E1 when referred to E20 panels.

Carbon fiber (CF)-reinforced plywood prepared with a perpendicular orientation shows that WA after 24 h is consistent [55]. After soaking, the thickness of the CF plywood at the top layer increased, possibly due to previously compacted and heat-pressed fibers. The sample containing cross-structure CF exhibited the maximum water absorption after 2 and 24 h. Water absorption in plywood with CF was the same after 2 and 24 h.

The water absorption tests in a study carried out by [56] on HDF glued with rice glue show that absorption diminishes as the amount of rice glue increases. Higher rice glue content causes the absorption to decrease; sample R10 shows this after 2 h at 226%, and sample R20 shows this after 2 h at 160% and 24 h at 179%. The reference sample absorbs the least water after 2 and 24 h, at 80% and 82%, respectively.

## 4. Conclusions

The alternative filler content significantly impacts the mechanical and physical characteristics of the produced plywood. It was found that when the amount of filler is increased to 10% by weight, the MOE rises. Additional increases in this content cause the MOE to drop significantly, producing findings lower than the samples’ reference values. The samples that had 5% by weight of filler had the best MOR and IB findings. This parameter decreased for both lower and larger quantities of the tested substance. According to water absorption research, calcium carbonate has a far lower capacity to absorb water than rye starch. This characteristic could be a significant factor in the plywood’s decreased swelling thickness. When eggshells were employed, the more eggshells there were, the less swelling of the plywood was seen. However, there was also a noticeable rise in water absorption at the same time.

While the study successfully demonstrates the potential of ground eggshells as an eco-friendly filler in plywood production, several limitations should be acknowledged. Firstly, the variability in the mechanical and physical properties observed across different filler concentrations suggests that factors such as the homogeneity of the adhesive mixture and the manual application method could have influenced the results. The lack of automation in adhesive application may have introduced inconsistencies, particularly in the uniformity of the bonding line and filler distribution. Secondly, while the environmental benefits of upcycling eggshells were discussed, a comprehensive life-cycle analysis (LCA) was not conducted. This would provide a clearer picture of the ecological advantages and potential trade-offs compared to conventional fillers. Addressing these limitations in future research would further validate the findings and strengthen the applicability of this innovative approach in sustainable wood composite manufacturing.

## Figures and Tables

**Table 1:** The varieties of tested composites.

Varieties of Tested Composites
Plywood with 0 pbw of filler (without added filler) (hereafter called E0) Plywood with 1 pbw ES as a filler (hereafter called E1) Plywood with 5 pbw ES as a filler (hereafter called E5) Plywood with 10 pbw ES as a filler (hereafter called E10) Plywood with 20 pbw ES as a filler (hereafter called E20) Plywood with 10 pbw rye flour (reference; hereafter called REF10)

## Data Availability

The original data presented in the study are openly available in RepOD at https://doi.org/10.18150/NP4QWZ.
